# Clinical‐Stage Nanatinostat Triggers Productive Epstein‐Barr Virus Lytic Replication

**DOI:** 10.1002/jmv.71077

**Published:** 2026-07-28

**Authors:** Ibukun A. Akinyemi, Travis M. Zeigler, Griffin H. Willman, Devshree Patel, Ayman El‐Guindy, Michael T. McIntosh, Sumita Bhaduri‐McIntosh

**Affiliations:** ^1^ Child Health Research Institute, Department of Pediatrics University of Florida Gainesville Florida USA; ^2^ Division of Infectious Diseases, Department of Pediatrics University of Florida Gainesville Florida USA; ^3^ Viracta Therapeutics Encinitas California USA; ^4^ AbVir Therapeutics California USA; ^5^ Department of Molecular Genetics and Microbiology University of Florida Gainesville Florida USA

**Keywords:** Epstein‐Barr virus, HDAC inhibitor, kick‐and‐kill, lymphoma, lytic cycle, lytic replication, nanatinostat, reactivation

## Abstract

A phase 1b/2 study of the all‐oral “kick‐and‐kill” combo nanatinostat + valganciclovir (nana‐val) showed a ~40% overall response rate in patients with relapsed/refractory Epstein‐Barr virus (EBV)‐associated lymphomas. Despite the promising results in these difficult‐to‐treat cancers, there is little known about the effects of nanatinostat on EBV and cell death. In this work we demonstrate that nanatinostat, an HDAC1/3 inhibitor, robustly induces the full EBV lytic cycle in a panel of EBV‐positive lymphoma and transformed cell lines (Burkitt, DLBCL, lymphoblastoid), spanning viral latency types and EBV genotypes. Nanatinostat drives lytic‐gene transcription across all kinetic classes, viral genome replication, and virion release, and kills cells at nanomolar concentrations. Although valganciclovir does not increase cytotoxicity, its ability to block viral DNA synthesis and virion production justifies its inclusion to prevent viral dissemination. Importantly, the two main metabolites of nanatinostat lack histone‐H3 acetylation activity and neither activate nor inhibit the EBV lytic phase, indicating that they are biologically inert with respect to EBV. These mechanistic insights clarify the clinical efficacy of nana‐val and support further development of kick‐and‐kill strategies for EBV‐driven malignancies.

## 1 Introduction

Epstein‐Barr virus (EBV)‐cancers present a significant global burden estimated at ~300,000 new cases and 137,000—208,000 deaths in 2020—translating to 2% of global cancer‐related deaths [1]. EBV contributes to several types of cancers of B and epithelial cell origin. EBV is associated with up to 15% of diffuse large B‐cell lymphomas (DLBCL), up to 30% of classical Hodgkin lymphoma (cHL), 30%–100% of T cell and NK cell lymphomas, 60%–80% of post‐transplant/immunodeficiency‐associated lymphoproliferative diseases/lymphoma (PTLD/LPD), and nearly 100% of endemic Burkitt lymphomas (BL). EBV also contributes to nasopharyngeal cell carcinoma and gastric carcinoma. While immunosuppression is a recognized risk factor and some EBV‐lymphomas arise in overtly immunosuppressed hosts, most occur in individuals with apparently healthy immune systems arguing against a systemic loss of EBV control in most cases. Reduction in immunosuppression, when possible, is an obvious intervention in some patients with EBV‐lymphomas. However, for most, therapeutic options consist of standard chemotherapy including R‐CHOP and when appropriate, Rituximab (anti‐CD20 antibody). That said, EBV‐lymphomas tend to be highly aggressive with 5‐year overall survival rates of only 20%–25% for DLBCL and peripheral T cell lymphoma and 2‐year overall survival rates of 44% for extranodal NK/T‐cell lymphoma [2, 3, 4]. Notably, the prognosis for patients with EBV‐positive lymphomas is significantly worse than for those with EBV‐negative lymphomas, with nearly 50% of patients with EBV‐positive DLBCLs experiencing refractory disease or relapse [5, 6, 7].

Although EBV‐associated lymphomas exhibit substantial molecular and immunologic heterogeneity, complicating efforts to identify shared host‐directed targets, the virus itself provides a selective and therapeutically actionable vulnerability. EBV infects more than 95% of the global population and persists lifelong in B lymphocytes in a latent state. During latency, only a limited set of viral genes is expressed, whereas the majority of genes required for the lytic phase remain epigenetically silenced. Periodically, through incompletely understood mechanisms, latency is disrupted and lytic gene expression is initiated. The lytic cycle begins with expression of the immediate‐early gene *BZLF1*, which encodes the master transcriptional activator ZEBRA, the protein that drives the latency‐to‐lytic switch. ZEBRA expression triggers a coordinated cascade of early lytic gene expression, viral genome replication, and ultimately virion assembly and release.

The vast majority of tumor cells in EBV‐associated lymphomas predominantly harbor the virus in its latent state. In experimental systems, lytic reactivation can be induced using pharmacologic or immunologic stimuli, including HDAC inhibitors (HDACi), DNA methyltransferase inhibitors, and IgG crosslinking. These observations led to development of the “kick‐and‐kill” strategy, in which pharmacologic induction of the lytic cycle (“kick”) promotes expression of the viral protein kinase that phosphorylates and activates a nucleoside analog (“kill”), enabling its incorporation into viral and host DNA, blocking genome replication, and triggering tumor cell death.

In a recent phase 1b/2 clinical trial, patients with diverse EBV‐associated lymphomas—including cHL, B‐Non‐Hodgkin lymphomas (NHL, including DLBCL), T/NK‐NHL, and LPD – were treated orally with the class I HDACi nanatinostat (targeting HDAC1 and HDAC3) in combination with the nucleoside analog valganciclovir [8]. Notably, 84% of participants had stage III‐IV disease, and 75% were refractory to their most recent therapy. Despite this heavy pretreatment and advanced disease burden, the overall response rate (ORR) among evaluable patients was 40%, with ORRs of 60% and 67% in patients with T/NK‐NHL and DLBCL, respectively. However, despite these promising outcomes, the biological mechanism responsible for the observed clinical responses has yet to be elucidated. Although assumed to, does nanatinostat indeed reactivate EBV, and if so, how does it compare to reactivation by well‐established lytic cycle triggers? Does nanatinostat‐mediated reactivation result in completion of the lytic phase or is the lytic phase abortive? Is the lytic reactivation “kick” by nanatinostat sufficient for tumor cell death or is the “kill” activity of valganciclovir also needed? Are metabolites of nanatinostat inert or do they contribute to EBV lytic reactivation?

In this report, we provide preclinical evidence that nanatinostat reactivates EBV across multiple EBV‐associated lymphoma models—including BL, DLBCL, and EBV‐transformed lymphoblastoid cell lines (LCLs)—irrespective of EBV type, latency program, or geographic origin. We find that nanatinostat, at nanomolar concentrations, drives the lytic cycle to completion and that while addition of Ganciclovir is essential to prevent the spread of the virus, the nucleoside's ability to add to lymphoma cell death is questionable. Furthermore, two main metabolites of nanatinostat lack the ability to acetylate histones, do not trigger viral reactivation, or interfere with nanatinostat's ability to reactivate EBV, confirming that the drug's breakdown products remain inert and do not spread the virus.

## 2 Results

### 2.1 Nanatinostat Triggers EBV Reactivation and Lytic Cycle Progress in EBV‐Lymphoma Cells, Irrespective of the Type of Viral Latency, Type of EBV, or Molecular Characteristics of the Lymphoma/Transformed Cells

Because nanatinostat was clinically evaluated across a spectrum of EBV‐positive B‐cell non‐Hodgkin lymphomas (B‐NHL), we selected cell lines that reflect the biological breadth of EBV‐associated B lymphomas. Although EBV persists predominantly in a latent state in these tumors, EBV‐positive lymphomas are molecularly diverse and exhibit distinct viral latency programs. BL, for example, is defined by translocation of the *MYC* oncogene to the immunoglobulin heavy‐ or light‐chain locus, resulting in constitutive c‐MYC overexpression. In EBV‐positive BL, viral gene expression patterns further stratify tumors into latency I or the less restricted Wp‐restricted latency. Akata and Mutu‐I cells represent latency I BL, whereas HH514‐16 cells harbor EBV with Wp‐restricted latency [9, 10, 11, 12]. Latency I tumors express the viral genome maintenance protein EBNA1 (from the Qp promoter) along with noncoding EBER transcripts and BARF0, but lack expression of the major viral oncoproteins EBNA2, LMP1, and LMP2. In contrast, Wp‐restricted BL—representing a substantial 15% of BL worldwide—contains EBV genomes with deletions in EBNA2 and part of EBNA‐LP [13]. These tumors express EBNA1, EBNA3A, EBNA3B, EBNA3C, and the viral BCL2 homolog BHRF1 from the Wp promoter. The selected BL lines also reflect geographic, molecular, and viral genetic diversity. HH514‐16 and Mutu‐I were derived from African patients and represent endemic BL, whereas Akata was derived in Japan and represents sporadic BL. In contrast to latency I BLs, Wp‐restricted BLs (e.g. HH514‐16) downregulate the germinal center‐associated marker BCL6 while upregulating markers of plasmacytoid differentiation including IRF4 and BLIMP1 [13]. Collectively, these lines harbor both major EBV types: type 1 (Akata and Mutu‐I) and type 2 (HH514‐16). Beyond BL, EBV persists in latency III in EBV‐transformed LCLs, which serve as models of PTLD/LPD and certain DLBCL; in latency III, all viral latent proteins are expressed [14]. Importantly also, qualitative and quantitative differences exist in the capacity of EBV to undergo lytic reactivation in response to distinct inducing agents, underscoring the need to evaluate therapeutic strategies across diverse latency contexts.

**Figure 1:**
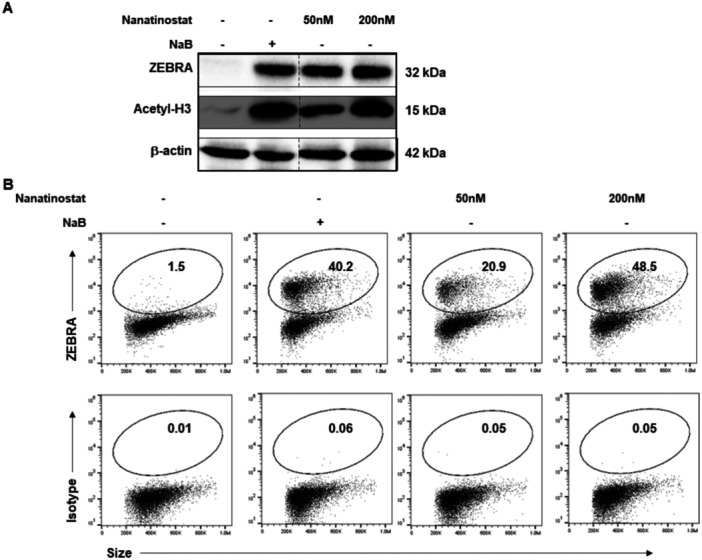
Nanatinostat induces expression of the EBV latent‐to‐lytic switch protein ZEBRA at levels comparable to a pan‐HDAC I and II inhibitor. EBV‐positive HH514‐16 BL cells were exposed to indicated concentrations of nanatinostat or sodium butyrate (NaB) for 24 h followed by immunoblotting with antibodies (A) and flow cytometric enumeration of ZEBRA positive, that is, lytic, cells (B).

**Figure 2:**
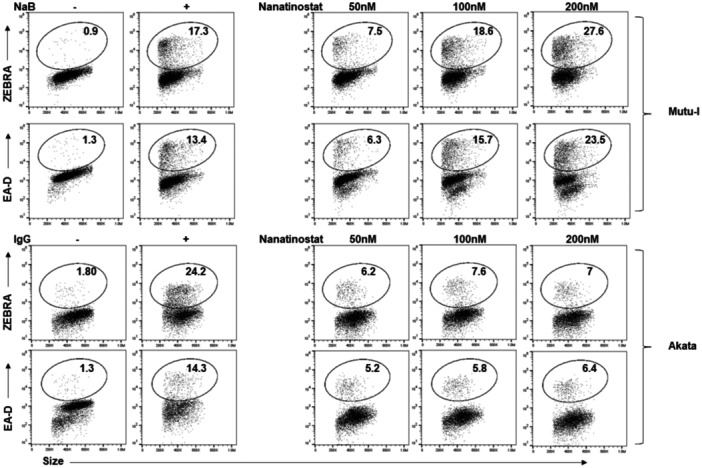
EBV reactivation and viral early gene expression in other BL cells point to general BL cell responsiveness to nanatinostat. EBV‐positive Mutu‐I and Akata BL cells were treated with NaB or nanatinostat for 24 h followed by quantitation of ZEBRA and EA‐D positive cells by flow cytometry; ZEBRA transactivates the viral gene *BMRF1* which encodes the early lytic gene product EA‐D that is essential for viral genome replication.

**Figure 3:**
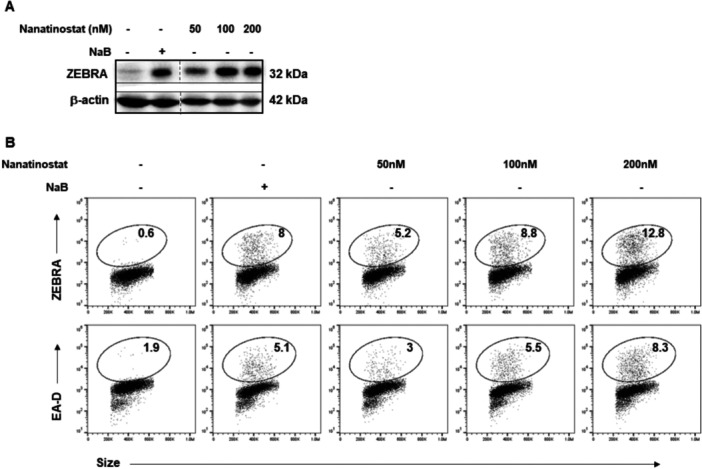
Lymphoblastoid cell lines, an important model for PTLD, respond to nanatinostat. LCLs were exposed to NaB or nanatinostat and 24 h later subjected to immunoblotting (A) and flow cytometry for enumeration of ZEBRA‐ and EA‐d‐positive lytic cells (B).

Bearing the above considerations in mind, we tested HH514‐16 cells in which EBV is tightly latent at baseline but can be robustly reactivated by lytic triggers. As shown in Figure 1A, the lytic agent sodium butyrate (NaB), an HDACi, and both 50 and 200 nM nanatinostat caused robust acetylation of histone‐H3 and expression of the lytic switch protein ZEBRA compared to uninduced cells. Like NaB, 200 nM nanatinostat caused reactivation in nearly half the cells (Figure 1B). While 50 nM and 200 nM nanatinostat caused similar levels of upregulation of ZEBRA by immunoblotting, 200 nM nanatinostat reactivated EBV in more than twice the number of cells compared to 50 nM nanatinostat, underscoring the importance of examining the number of lytic cells (Figure 1B). When applied to Mutu‐I cells, nanatinostat demonstrated a dose dependent increase in the fraction of ZEBRA‐positive lytic cells with most of them also progressing to express EA‐D (Figure 2); EA‐D is the DNA polymerase processivity factor that is expressed by the early lytic gene *BMRF1*, transcriptionally activated by ZEBRA. In Akata cells, the effect of nanatinostat reached a plateau at 50 nM with most of the lytic cells also expressing EA‐D (Figure 2). We note that EBV in Akata cells is known to be primarily reactivated by cross‐linking cell surface IgG [15]. Similarly, nanatinostat reactivated EBV in LCLs in a dose‐dependent manner, with the lytic cycle also progressing to EA‐D expression in most of the cells (Figures 3A,B); notably, LCLs are generally difficult to induce into the lytic phase. Of relevance also, for the flow cytometry results in Figures 2 and 3, cells staining for ZEBRA and EA‐D are not mutually exclusive because EA‐D expression depends on ZEBRA, and therefore, EA‐D expressing cells must also express ZEBRA. Overall, nanatinostat robustly reactivates EBV to drive lytic cycle progress in lymphoma/transformed cells regardless of the geographic origin or molecular characteristics of the lymphoma, EBV latency type, and type of EBV.

### 2.2 A Short Window of Exposure to Nanatinostat Effectively Triggers EBV Reactivation and Lymphoma Cell Death Without additional toxicity From a Nucleoside Analog

**Figure 4:**
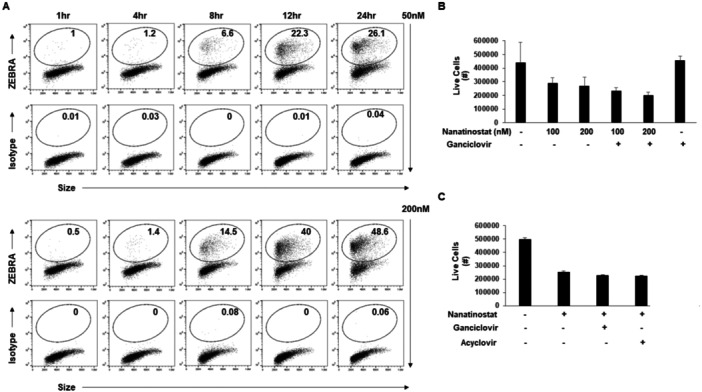
A 12‐h exposure to nanatinostat is sufficient to optimally trigger reactivation of EBV and kill EBV‐cancer cells. HH514‐16 cells were exposed to nanatinostat for increasing lengths of time followed by wash‐off and evaluation of ZEBRA‐positive lytic cells by flow cytometry (A). Cells were exposed to nanatinostat for 12 h (B) or 48 h (C), washed and resuspended at 500,000/ml, exposed to a nucleoside analog, and harvested at 72 h from the start of nanatinostat exposure for evaluation of live cells by propidium iodide staining and flow cytometry; total cell numbers are presented. Experiments in B and C are representatives of biological duplicates; error bars, SD of technical triplicates; statistical significance is not shown as *N* = 2.

The predicted mechanism of kick‐and‐kill therapy suggests that the lytic trigger reactivates the virus with the nucleoside analog killing the cells by blocking viral and cellular genome replication. To address the contribution of each therapeutic component to cell death, we first determined the shortest exposure needed for nanatinostat to reactivate EBV. Further, because we were interested in examining lymphoma cell death, we chose HH514‐16, among the most tightly latent, the most responsive (to nanatinostat), and the “hardiest” of the lymphoma lines. Compared to Latency I, Wp‐restriction confers a substantial anti‐apoptotic phenotype—mediated by Wp‐driven expression of the viral BCL2 encoded by *BHRF1* with contributions also from the EBNA3 proteins and *BHRF1* miRNAs [16, 17, 18, 19]. Figure 4A is a wash‐off experiment showing that 12 h of exposure to 200 nM nanatinostat was sufficient for near‐maximal numbers of cells to support EBV reactivation.

Using 200 nM and a lower concentration of 100 nM for 12 h, we examined the effect of nanatinostat alone versus nanatinostat plus Ganciclovir on cell killing; here, Ganciclovir was added for 60 h following nanatinostat wash‐off. We found that nanatinostat alone was sufficient to cause near‐maximal cell death with little to no added benefit when Ganciclovir was used in addition (Figure 4B). As expected, Ganciclovir alone had no effect on lymphoma cell death. To assess if longer exposure to nanatinostat enhanced cell killing, we washed‐off nanatinostat after 48 h of exposure followed by exposure to Ganciclovir or Acyclovir (another anti‐herpesvirus nucleoside analog with a similar mechanism of action) for the next 24 h (Figure 4C). These results, mirroring those shown in Figure 4B, also showed that nanatinostat was sufficient to cause cell death without added benefit from longer treatment or addition of a nucleoside analog. These experiments provide support for the use of intermittent nanatinostat dosing in the clinical trial and indicate that valganciclovir likely did not contribute to lymphoma cell death; oral valganciclovir, used in the clinical trial, is a prodrug that is rapidly converted into the active antiviral agent Ganciclovir in the body.

### 2.3 Nanatinostat Drives the EBV Lytic Phase to Completion Making it Necessary to Use a Nucleoside Analog to Prevent Viral Dissemination

**Figure 5:**
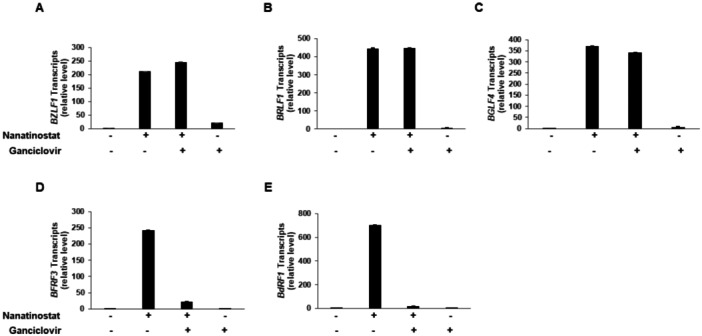
Nanatinostat promotes robust transcription of EBV lytic genes of all kinetic classes. HH514‐16 cells were treated with 100 nM nanatinostat for 12 h followed by washing and exposure to Ganciclovir for another 24 h. RT‐qPCR was performed to quantitate immediate early (*BZLF1*, *BRLF1;* A, B), early (*BGLF4*; C), and late (*BFRF3*, *BdRF1*; D, E) lytic transcripts. Representatives of biological duplicates are shown; error bars, SD of technical triplicates; statistical significance is not shown as *N* = 2.

**Figure 6:**
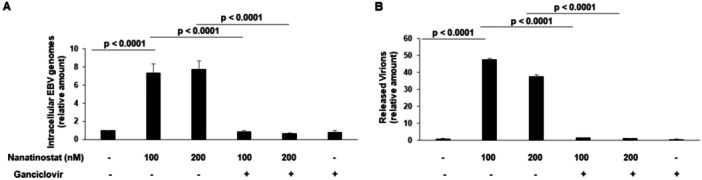
Following lytic reactivation, nanatinostat drives the EBV lytic cycle to completion. After exposure to nanatinostat for 12 h, HH514‐16 cells were washed and placed back in culture in the presence or absence of Ganciclovir, followed by harvest 24 h later for quantitation of intracellular EBV genomes by *BALF5* qPCR (A). To quantify encapsidated virions, culture supernatants were harvested at 72 h from the start of nanatinostat exposure and analyzed by *BALF5* qPCR following DNase treatment (B). Error bars, SEM of biological triplicates.

The ultimate objective of the EBV lytic cycle is the production and release of infectious progeny virions capable of infecting new B cells and thereby contributing to viral persistence and tumorigenesis. A complete lytic program proceeds through a tightly coordinated cascade of events initiated by expression of the immediate‐early transactivators ZEBRA and RTA (encoded by *BRLF1*). Their activation drives early lytic gene expression, viral genome replication, genome packaging, virion assembly, and ultimately virion release. Importantly, lytic reactivation can also be abortive or incomplete, with interruption at various stages of the cascade. The point at which the lytic program terminates determines cellular fate and whether infectious virions are produced, thereby influencing both tumor cell survival and the potential for viral spread. We therefore examined if nanatinostat drove transcription of a) immediately‐early genes *BZLF1* and *BRLF1*, both of which are required for completion of the lytic phase [20], b) the early lytic gene *BGLF4*, the kinase needed to activate Ganciclovir in the cells, and c) late lytic genes *BFRF3* and *BdRF1* that contribute to virus assembly and release. We found that nanatinostat drove expression of each of these transcripts while Ganciclovir alone did not. When added to nanatinostat, Ganciclovir did not impact the expression of the immediate‐early and early genes but blocked expression of the late lytic genes (Figure 5A–E). These results suggested that as expected, Ganciclovir successfully blocked viral genome replication since late gene expression temporally follows and depends on genome replication. Supporting these findings, we also found that nanatinostat addition resulted in viral genome replication that was blocked when Ganciclovir was added (Figure 6A).

While nanatinostat promoted expression of all kinetic classes of lytic genes and viral genome replication, the lytic phase could still be interrupted post genome replication such that no virions are released. That, however, was not the case – as shown in Figure 6B, nanatinostat‐mediated EBV reactivation culminated in the release of encapsidated virions which could be effectively suppressed by Ganciclovir. Collectively, nanatinostat reactivates EBV to drive the complete lytic phase, necessitating the use of a nucleoside analog to ensure that no infectious/transforming virus is released during kick‐and‐kill therapy.

### 2.4 Important Metabolites of Nanatinostat are Biologically Inert With Respect to EBV Lytic Cycle Induction

**Figure 7:**
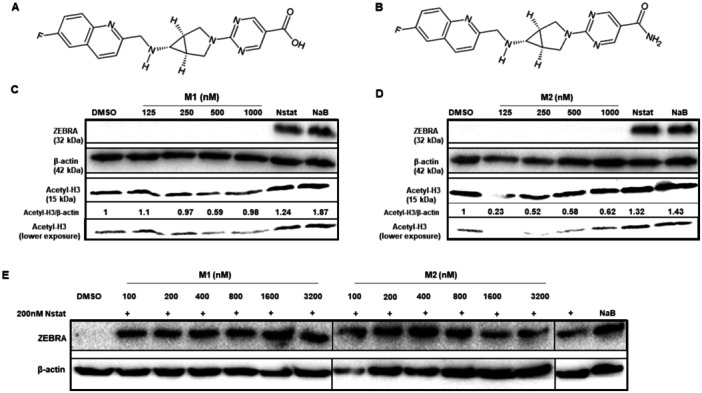
Two major metabolites of nanatinostat fail to hyperacetylate histone‐H3, reactivate EBV, or inhibit nanatinostat‐mediated EBV reactivation. Structures of two main metabolites of nanatinostat M1 (A) and M2 (B) are shown. HH514‐16 cells were exposed to increasing concentrations of M1 or M2 alone (C and D, respectively) or M1 vs M2 with nanatinostat (E) for 24 h followed by immunoblotting.

We next investigated whether the clinical benefits observed in patients treated with nanatinostat were attributable solely to the parent compound or also to active metabolites that may contribute to EBV reactivation. Pharmacokinetic studies of nanatinostat in human hepatocytes identified seven metabolites. Of these, M2 was the predominant species (52.16%) followed by M1 (18.69%), while the remaining five metabolites were each present at levels below 0.1%; the parent compound constituted 29.01%. M1 represents a hydrolysis product and M2, an N‐hydroxyl reduction product of nanatinostat. We tested the two main metabolites of nanatinostat, M1 and M2 (Figure 7A,B). As shown in Figure 7C,D, unlike NaB and nanatinostat, neither M1 nor M2 was able to induce histone‐H3 hyperacetylation. M1 and M2 also did not reactivate EBV even when used at high concentrations reaching 1000 nM. Furthermore, the presence of high concentrations of M1 and M2 did not impair nanatinostat's ability to reactivate EBV (Figure 7E). These results indicate that the effect of nanatinostat on the EBV lytic cycle tightly correlates with its HDAC inhibitory function and that its two main metabolites are inert with regard to HDAC inhibition and EBV reactivation, also allaying concerns about continued off‐target effects of the metabolites on host gene expression.

### 2.5 Nanatinostat Reactivates EBV in DLBCLs Explanted From Non‐AIDS Patients

**Figure 8:**
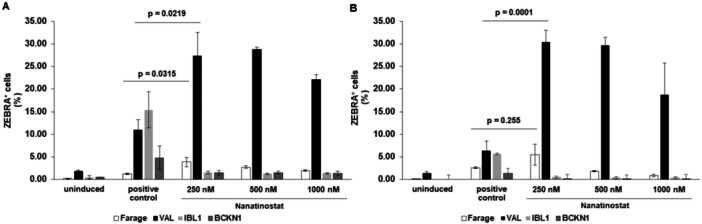
Nanatinostat effectively reactivates EBV in DLBCL cell lines derived from non‐AIDS patients but fails to do so in lines derived from AIDS patients. EBV‐positive DLBCL lines derived from non‐AIDS patients (Farage and VAL) and AIDS patients (IBL‐1 and BCKN‐1) were exposed to known lytic cycle inducing agents (NaB+TPA for Farage, anti‐IgG for the other lines) or increasing concentrations of nanatinostat and harvested after 24 h (A) or 48 h (B) for enumeration of lytic cells by ZEBRA staining and flow cytometry. Error bars, SEM of biological triplicates.

In the nanatinostat trial, the ORR in patients with DLBCL was 67% with a complete response (CR) of 33%. Based on our observation that nanatinostat successfully induces the EBV lytic cycle by turning ZEBRA on, we assessed nanatinostat's ability to reactivate EBV in DLBCL lines. Of the four available EBV‐positive DLBCL lines, two were derived from HIV‐negative patients (Farage and VAL lines) and two from AIDS patients (IBL‐1 and BCKN‐1 lines). We tested a range of concentrations of nanatinostat for 24 and 48 h and compared their ability to reactivate EBV alongside lytic triggers known to reactivate EBV in each cell line (i.e. positive control) [21]. Overall, as shown in Figure 8A,B, 250 nM nanatinostat performed best in both Farage and VAL cells with the percent lytic cells significantly exceeding the positive control lytic cycle inducing agent/combination at one or both time points. In contrast, nanatinostat failed to induce the lytic phase in the two DLBCL lines derived from AIDS patients. Although there were only two AIDS patients with DLBCLs in the clinical trial, neither responded to therapy with nanatinostat. Thus, the complete and partial responses observed in DLCBL patients treated with nanatinostat are supported by nanatinostat's ability to reactivate EBV in DLBCL lines derived from HIV‐negative patients.

## 3 Discussion

As promising as the report by Haverkos et al. [8] on the phase 1b/2 study of an oral kick‐and‐kill approach for EBV‐lymphomas was, the lack of preclinical evidence defining nanatinostat's effects on EBV biology represents a critical gap in our mechanistic understanding of this therapeutic strategy. While it is standard practice to follow EBV loads in patients suspected or confirmed to have EBV‐lymphomas, the relative contribution from viral genomes in latently‐infected/tumor cells versus those from lytically reactivated cells is difficult to ascertain. Therefore, plasma EBV DNA loads from the subjects in the clinical study were unable to inform on nanatinostat's contribution to EBV reactivation and progress of the lytic phase. This is an important mechanistic distinction since HDACi have been shown to inhibit proliferation and tumor progression of several solid tumors and hematologic malignancies [22, 23]. These anti‐tumor effects of HDACi in EBV‐unrelated cancers are thought to be mediated via epigenetic changes, altered chromatin structure, ultimately changing gene transcription to effect cell cycle arrest and apoptosis [22, 23, 24]. In the context of EBV‐lymphomas, however, our findings demonstrate that nanatinostat is a powerful driver of the EBV lytic program across diverse lymphoma subtypes and establish that the virus present within these tumors represents a therapeutically targetable vulnerability.

Although nanatinostat is an HDACi, it is chemically distinct from the frequently used HDACi NaB that is a short chain fatty acid. It is, however, similar to the lytic cycle inducing agents trichostatin A and SAHA (Suberanilohydroxamic acid), in that they are hydroxamic acid derivatives. How lytic triggers including HDACi reactivate EBV remains incompletely understood but several complementary mechanisms contribute to remove repressive forces on lytic promoters while simultaneously exerting forces to activate those promoters, in particular the *BZLF1* promoter. These include upregulation of cellular immediate early genes such as *TXNIP* to activate the inflammasome which disrupts host repressor complexes (e.g. NuRD‐HDAC associated KRAB‐ZFP) at *BZLF1* and other lytic promoters, recruitment of host transcription factors such as AP‐1 and XBP1 to activate the *BZLF1* promoter, increasing histone acetylation to relax the chromatin at lytic promoters, and alteration of higher order chromatin structure [25, 26, 27, 28, 29, 30, 31, 32]. As mentioned previously, EBV‐lymphoma subtypes are distinct in their molecular characteristics, the type of latency that EBV exhibits and therefore the range of EBV latency genes expressed, the EBV type, the geographic location, and the immune status of the host. Given this diversity, not surprisingly, specific EBV‐lymphoma lines are only responsive to some lytic triggers and not others; moreover, in certain lines such as LCL, EBV is particularly difficult to reactivate. Yet, several of the lymphoma lines studied here and representative of the above EBV‐lymphoma subtypes respond to nanatinostat.

Lytic reactivation induced by nanatinostat reflects a fundamental feature of EBV biology: disruption of latency occurs in only a subset of infected cells. This partial permissiveness to lytic stimuli is a well‐recognized characteristic of EBV‐positive lymphoma cell lines and primary tumors and is consistent with the broader biology of herpesviruses [33]. Whether a minimal fraction of latently infected tumor cells must undergo EBV reactivation to secure clinical benefit remains uncertain. This threshold likely hinges not only on the proportion of cells entering the lytic cycle but also on host immune competence and the ability of viral antigens expressed during the lytic phase to elicit a robust anti‐tumor immune response. While it is tempting to correlate the extent of lytic reactivation in cell lines with clinical responses in lymphoma patients, an important consideration is that our experiments were performed with a single administration of nanatinostat. By contrast, in the nana‐val clinical trial, patients received nanatinostat up to twice a day every day for 28 days or on a 4 days on/3 days off schedule for 28 days in the RP2D expansion cohort. A second consideration is the important role played by the immune response that is elicited by viral antigens expressed during the lytic phase—this is lacking in the in vitro experiments. Given these key differences between our in vitro experiments and the clinical trial, it is not possible (or appropriate) to directly equate the efficiency of lytic reactivation with clinical responses in patients.

Although no data from patients with BL was reported in the kick‐and‐kill trial, individuals with endemic BL remain in urgent need of new therapeutic options because access to tertiary and specialized medical care, including dialysis support, is often limited in endemic regions. Such resources are frequently required to manage life‐threatening complications of current intensive therapies, particularly tumor lysis syndrome. In this context, our experiments with BL cell lines suggest that kick‐and‐kill approaches may provide a valuable bridge to standard chemotherapy regimens, which can be effective but typically depend on specialized supportive care infrastructure.

BL cells also harbor EBV in latency I, the most transcriptionally restricted latency program, characterized by expression limited to viral *EBNA1* and viral non‐coding RNAs. As such, BL represents a stringent model for testing pharmacologic lytic reactivation. Nanatinostat's ability to induce lytic reactivation in latency I cells thus provides strong mechanistic evidence that the drug can overcome even highly repressive chromatin environments. This supports the biological plausibility of inducing lytic reactivation across the broader spectrum of EBV latency programs encountered clinically, including latency II (e.g., Hodgkin lymphoma, DLBCLs, and NK/T cell lymphomas) and latency III (e.g., immunodeficiency‐associated lymphoproliferative disorders and some DLBCLs).

EBV lytic cycle induction is not binary; it exists along a spectrum from early/abortive activation to full productive replication. Where a tumor cell falls on this spectrum has important consequences for the efficacy and safety of therapeutic kick‐and‐kill approaches. Therefore, the choice of a lytic trigger is a key factor. With agents that induce a complete/productive lytic cycle, potential advantages include expression of viral kinases that activate nucleoside analogs, viral DNA replication that provides the substrate for chain‐terminating nucleoside analogs, viral cytopathic effects that may directly contribute to tumor cell death, and lytic antigen expression that may enhance immune‐mediated clearance. On the other hand, release of infectious virions resulting in new infection of bystander B cells and possible viral dissemination especially in immunocompromised patients and inflammatory consequences of widespread lytic replication are important risks. As for agents that activate an abortive/incomplete lytic cycle, potential advantages include expression of a limited set of early lytic genes, limited or absent viral DNA replication, and lack of late gene expression and virion production. Potential limitations include insufficient viral kinase levels resulting in suboptimal nucleoside analog activation, lack of incorporation of chain‐terminating drugs due to reduced viral DNA replication, and survival of tumor cells if the lytic program stalls early. That said, our observations from Figure 4 indicate that the lytic phase is sufficient to kill cells even when lytic cycle progress is aborted upstream of DNA replication. Overall, for therapeutic efficacy, three thresholds likely matter: expression of *BZLF1* (and *BRLF1*/RTA), early gene expression especially viral kinases, and sufficient viral DNA synthesis to enable toxic nucleoside analog incorporation. By inducing a full lytic cycle, nanatinostat falls in the first category of lytic cycle trigger; it can reactivate EBV at low concentrations.

In the nanatinostat trial, the function of Ganciclovir was predicted to be two‐fold: a) cause cell death by chain termination of replicating viral and host DNA and b) prevent dissemination of infectious virions which can be deleterious to the host. While it was not possible to distinguish between these mechanisms from the clinical study, our experiments indicate that Ganciclovir's primary function was to block viral genome replication, thereby preventing the release of infectious virions. Viral cytopathic effects (and possibly immune activation in patients) contributed to tumor cell death. Importantly, also, Ganciclovir did not block nanatinostat‐mediated cell death in our study. In using the nanatinostat‐valganciclovir combination, the trial was able to accomplish robust lytic cycle activation to trigger tumor cell death, drive expression of early lytic genes including viral kinases to activate Ganciclovir, and avoid extensive virion release. No disease flares or fulminant EBV (or even cytomegalovirus, CMV) reactivations were noted in the clinical study.

Drug metabolites warrant careful evaluation, as they may augment, attenuate, or otherwise alter the activity of the parent compound. Our study demonstrates that the two principal metabolites of nanatinostat lack HDAC inhibitory activity. Consistent with this, neither metabolite induced EBV lytic reactivation. Importantly, they also did not interfere with nanatinostat's ability to trigger the lytic program. Together, these findings indicate that the observed effects on EBV reactivation and tumor cell death are attributable to the parent compound itself. Together, our data mechanistically substantiate the clinical responses observed in patients with heterogeneous EBV‐associated lymphomas treated with oral nanatinostat‐valganciclovir. As interest in kick‐and‐kill approaches for EBV‐driven cancers accelerates, these findings provide critical biological validation to guide rational therapeutic optimization and next‐generation clinical trials.

## 4 Materials and Methods

### 4.1 Cell Lines and Chemical Treatment of Cells

EBV‐positive BL cell lines (HH514‐16—a kind gift from Dr. George Miller, Yale University; Mutu‐I—a kind gift from Dr. Erik Flemington, Tulane University; Akata—a kind gift from Dr. Benjamin Gewurz, Harvard Medical School, Cambridge, MA), EBV‐transformed lymphoblastoid cell lines (LCL) generated as previously described [34], and EBV‐positive DLBCL cell lines (VAL—a kind gift from Dr Izidore Lossos, University of Miami and Farage—a kind gift from Dr Eric C. Johannsen, University of Wisconsin) were maintained in RPMI 1640 medium supplemented with 10% fetal bovine serum and 1% penicillin/streptomycin. EBV‐positive IBL‐1 and BCKN‐1 cells (kind gifts from Dr Ethel Cesarman, Weill Cornell Medical College) were maintained in RPMI 1640 medium supplemented with 20% fetal bovine serum and 1% penicillin/streptomycin. All cell lines were cultured in a 37°C incubator with 5% CO2.

HH514‐16, Mutu‐I and LCL cells were sub‐cultured at 5 × 10^5^ cells/mL and treated with 3 mM sodium butyrate/NaB (303410, Sigma‐Aldrich) and harvested 24 h later. Akata cells were treated with Rabbit anti‐human IgG (1:200, A042301‐2; Dako/Agilent), Farage (3 mM NaB + 20 ng/ml of 12‐O‐Tetradecanoylphorbol‐13‐Acetate (TPA, 4174, Cell Signaling), IBL‐1, BCKN‐1, VAL (200 μg/ml AffiniPure F(ab′)2 fragment goat anti‐Human IgG (H + L) (109‐006‐003, Jackson ImmunoResearch). As indicated, cells were treated with different concentrations (50 nM, 100 nM, 200 nM) of nanatinostat with or without 40 uM Gancyclovir/GCV (461710010, Acros Organics).

### 4.2 Extraction of RNA and DNA

RNA and DNA were isolated from cells using a Purelink RNA Mini kit (12183025, Invitrogen) and Genomic DNA mini kit (K182002, Invitrogen), respectively following manufacturer's instructions. RNA and DNA were quantitated using a NanoDrop instrument (Thermo Scientific).

### 4.3 RT‐qPCR

Relative transcript levels of selected viral genes were quantified using gene‑specific primers as previously described [30]. Briefly, 1 µg of total RNA was used for first‑strand cDNA synthesis with MuLV reverse transcriptase (M0253L, New England Biolabs) following the manufacturer's protocol and amplified with SYBR Green PowerUP Master Mix (A25778, Life Technologies) on a QuantStudio 3 thermocycler (Thermo Fisher Scientific). Data were analyzed using the ΔΔCT method. PCR primers included the following: 5'GTAACCCGTTGAACCCCATT3’ (forward) and 5'CCATCCAATCGGTAGTAGCG3’ (reverse) for 18S rRNA; 5'TTCCACAGCCTGCACCAGTG3’ (forward) and 5'GGCAGAAGCCACCTCACGGT3’ (reverse) for *BZLF1*; 5'ATGGCTGCTTCCTCCTTCTGG3’ (forward) and 5'CGAGGCAAGTCATCTGTTGG3’ (reverse) for *BRLF1*; 5'CGGTTTGAGCACCCTCATCT3’ (forward) and 5'GGCAAACGTGTAGGAGGTCA3’ (reverse) for *BGLF4*; 5'AACCAGAATAATCTCCCCAATG3’ (forward) and 5'CGAGGCACCCCAAAAGTC3’ (reverse) for *BFRF3*; 5'AGGAGGATGAAGAAGGCGGG3’ (forward) and 5'GCCTGTAGCGTGTGCTGTAA3’ (reverse) for *BdRF1*.

### 4.4 qPCR to Quantify Cell‐Associated Viral Genomes and Released Viral Genomes

Genomic DNA isolated from the cells and relative released viral genomes were quantified as previously described [30, 35] by qPCR targeting the EBV *BALF5* locus using the following primers: forward 5′‑CGTCTCATTCCCAAGTGTTTC‑3′ and reverse 5′‑GCCCTTTCCATCCTCGTC‑3′. To measure released EBV particles, equal volumes of culture supernatants were treated with DNase prior to qPCR analysis using *BALF5*‑specific primers. Relative EBV genome copy numbers were determined by comparison to a standard curve generated with the EBV BACmid p2089.

### 4.5 Antibodies

Antibodies used include mouse anti‐ZEBRA antibody clone BZ1 (sc‐53904, Santa Cruz Biotechnology), mouse anti‐EA‐D Ab (MAB8186, EMD), rabbit anti‐H3Ac (39040, Active Motif; clone D5E4, 8173S, Cell Signaling Biotechnology), mouse IgG (557273, BD Biosciences), goat anti‐mouse conjugated to FITC (F0257, Sigma), mouse anti‐β‐actin Ab (AC‐15, Sigma), Phycoerythrin (PE) conjugated goat anti‐mouse IgG (sc‐3738, Santa Cruz), HRP‐conjugated goat anti‐mouse IgG(H + L) (626520, Thermo Fisher Scientific), and HRP‐conjugated goat anti‐rabbit IgG(H + L) (31460, Thermo Fisher Scientific).

### 4.6 Immunoblot Analysis

Immunoblotting was performed as previously described [36]. Briefly, cells were lysed in RIPA buffer, and clarified lysates were resolved by 10% SDS–polyacrylamide gel electrophoresis, transferred to nitrocellulose membranes, and probed with the indicated antibodies.

### 4.7 Live/Dead Cells Assay

Propidium iodide (PI) staining was performed as previously described to quantify live and dead cells in unfixed samples [37]. Briefly, 2 × 10^5^ cells were seeded in 24‑well plates in medium containing the indicated concentrations of inhibitors or dimethyl sulfoxide (DMSO). Cells were pelleted and resuspended in fresh medium with inhibitors or DMSO every 48 h. At 24‑h intervals, cells were collected and stained with 1 µg/mL PI (P4864, Sigma‑Aldrich) without fixation or DNA denaturation. Live (PI‑negative) and dead (PI‑positive) populations were distinguished by flow cytometry and total number of live cells calculated.

### 4.8 Flow Cytometry

To measure the percentage of lytic cells after exposure to different chemicals, cells were stained as previously described [35]. Briefly, after exposure to the compounds, cells were fixed with BD Cytofix/Cytoperm solution (554722, BD Bioscience) at room temperature for 15 min, washed with 1X BD Perm/Wash buffer (554723, BD Bioscience), and incubated with indicated primary antibodies for 1 h at room temperature. After washing, cells were further incubated with corresponding secondary antibodies for another hour at room temperature, washed, and then subjected to flow cytometry. Flow cytometry data were acquired on a ThermoFisher Attune NxT Acoustic Focusing Cytometer. Cells were gated by comparing fluorescence profiles to cells stained with the corresponding isotype control antibody and analyzed using FlowJo software (Version 10).

### 4.9 Statistical Analysis

Differences among groups were analyzed using an unpaired, two‑tailed Student's t‑test. Statistical significance was defined as *p* < 0.05. Data are presented as the mean ± standard error of the mean (SEM) from at least three independent experiments unless otherwise indicated. For representative experiments from biological duplicates (Figures 4 and 5), standard deviation (SD) of technical triplicates are shown without *p* values.

## Conflicts of Interest

The authors have declared that no competing interest exists because Viracta Therapeutics has ceased to exist as a company. Furthermore, while Viracta was involved in the study design and provided the drug and its metabolites, they were not involved in the performance of experiments or analysis of data.

## Data Availability

All data supporting the conclusions are presented in the article. There are no large datasets associated with this work.
